# Supplementary material for: Fault-tolerant operation of a logical qubit in a diamond quantum processor
Source: Nature. 2022 May 5;606(7916):884–9. doi: 10.1038/s41586-022-04819-6 (PMC9242857; doi:10.1038/s41586-022-04819-6)
Supplement: Supplementary file 1 — This file contains eight sections: System Hamiltonian; Experimental setup; Experimental sequence; Parameters of the nuclear-spin qubits; Flag fault-tolerant quantum error correction; Proof of fault tolerance of the preparation scheme; Error distribution in the prepared state; Pseudocode and references. [file 41586_2022_4819_MOESM1_ESM.pdf]

nature portfolio

https://doi.org/10.1038/s41586-022-04819-6

Supplementary information

Fault-tolerant operation of a logical qubit in a diamond quantum processor

In the format provided by the authors and unedited

Supplementary Information for "Fault-tolerant operation of a logical qubit in a diamond quantum processor"

CONTENTS

I. System Hamiltonian 2

II. Experimental setup 3

III. Experimental sequence 3
A. NV preparation and initialization 6
B. Two-qubit gates 7
C. Phase synchronization and pulse timing 8
D. Initialization and final readout of the data qubits 9
E. Cross-phase calibrations 10
F. Circuit compilation 11
G. Echo sequences for the data qubits 11

IV. Parameters of the nuclear-spin qubits 14

V. Flag fault-tolerant quantum error correction 15
A. Stabilizer measurement with flag 17

VI. Proof of fault-tolerance of the preparation scheme 17

VII. Error distribution in the prepared state 20

VIII. Pseudocode 22

References 29

I. SYSTEM HAMILTONIAN

The Hamiltonian describing the physical system of an NV centre and the surrounding nuclear-spin qubits can be approximated as [1]:

$$H = H_e + H_N + H_C + H_{eN} + H_{eC} + H_{CC}, \tag{S1}$$

where H_e is the NV electron spin Hamiltonian; H_N is the Hamiltonian of the ^{14}N nuclear spin, H_c is the Hamiltonian of the ^{13}C spins, H_{eN} is the hyperfine interaction between the electron spin and the host ^{14}N nuclear spin, H_{eC} is the hyperfine interaction between the electron spin and the ^{13}C nuclear spins, and H_{CC} is the coupling between the nuclear-spin qubits.

Electron spin. The NV electron spin ground state is a spin triplet ($S = 1$) and its Hamiltonian can be approximated as (neglecting second order spin-orbit coupling) [2]:

$$H_e = \Delta_{\mathrm{ZFS}} S_z^2 + \gamma_e \mathbf{B} \cdot \mathbf{S}, \tag{S2}$$

where $\Delta_{\mathrm{ZFS}} \approx 2.88$ GHz is the zero field splitting, $\gamma_e \approx 2.8$ MHz/G is the electron gyromagnetic ratio, $\mathbf{B} = (B_x, B_y, B_z)$ is the magnetic field vector, and $\mathbf{S} = (S_x, S_y, S_z)$ are the electron spin-1 operators. The spin states $m_s = 0$ and $m_s = \pm 1$ are split by Δ_{ZFS} at zero applied magnetic field. In this work, we apply an external magnetic field of ~ 403 G which lifts the degeneracy of the $m_s = \pm 1$ states due to the Zeeman term. We define our electron-spin qubit between the states $m_s = 0$ ($|0\rangle$) and $m_s = -1$ ($|1\rangle$).

Nitrogen spin. The ^{14}N nuclear-spin Hamiltonian H_N and its interaction with the electron spin H_{eN} can be written as:

$$H_N + H_{eN} = -Q_N I_{N,z}^2 + \gamma_N \mathbf{B} \cdot \mathbf{I}_N + \mathbf{S} \cdot \mathbf{A}_N \cdot \mathbf{I}_N, \tag{S3}$$

where $Q_N \approx 4.98$ MHz is the quadrupole splitting (splitting the energy of the nitrogen-spin states $m_I = 0$ and $m_I = \pm 1$ at zero magnetic field) [2], $\gamma_N = 0.3077$ kHz/G is the nitrogen-spin gyromagnetic ratio, $\mathbf{I}_N = (I_{N,x}, I_{N,y}, I_{N,z})$ are the spin-1 operators for the ^{14}N nuclear spin, and $\mathbf{A}_N$ is the hyperfine tensor describing the electron-^{14}N interaction. Note that under the secular approximation, H_{eN} can be approximated as $A_{\parallel} \hat{S}_z \hat{I}_{N,z}$, where $A_{\parallel} = 2.16$ MHz is the parallel component of the hyperfine interaction.

Carbon-13 spins. The Hamiltonian for the ^{13}C spins H_c and their hyperfine interaction with the electron-spin H_{ec} can be written as:

$$H_C + H_{eC} = \gamma_C \sum_i \mathbf{B} \cdot \mathbf{I}_i + \sum_i \mathbf{S} \cdot \mathbf{A}_i \cdot \mathbf{I}_i, \tag{S4}$$

where $\gamma_C = 1.0705$ kHz/G is the carbon-spin gyromagnetic ratio, $\mathbf{I}_i = (I_{i,x}, I_{i,y}, I_{i,z})$ are the spin-1/2 operators for the ^{13}C nuclear spins, and $\mathbf{A}_i$ is the hyperfine tensor describing the electron-^{13}C interaction. Under the secular approximation, H_{eC} can be approximated as $\sum_i A_{\parallel} \hat{S}_z \hat{I}_{i,z} + A_{\perp} \hat{S}_z \hat{I}_{i,x}$, where $A_{\parallel}$ and $A_{\perp}$ are the hyperfine interaction components parallel and perpendicular to the applied magnetic field.

Nuclear-nuclear interactions. In addition to their interactions with the NV electron spins, the nuclear-spin qubits interact with each other via a weak dipolar coupling. For the work presented here, H_{CC} can be approximated as

$$H_{CC} = \sum_{i,j} C_{ij} \hat{I}_{i,z} \hat{I}_{j,z}, \tag{S5}$$

where C_{ij} are the dipolar spin-spin couplings, slightly modified due to the presence of the electron spin [3], and have been characterised for the system used here (Supplementary Table 5).

II. EXPERIMENTAL SETUP

Our setup consists of five main parts:

1. Control electronics: used to run the control cycle and to communicate between different components of the setup.
2. Lasers and optics: used for initialization and readout of the NV electron spin.
3. Microwave and RF: used for manipulating the spin states of the electron and nuclear spins.
4. External magnetic field: to create controllable energy splittings through the Zeeman effect.
5. Cryogenics: to cool down the diamond sample to 3.7 K, enabling long electron spin coherence times (> 1 s) and spin relaxation times (> 1 hour) [4].

See Supplementary Fig. 1 for a detailed sketch of the main components of the setup and how they communicate with each other.

III. EXPERIMENTAL SEQUENCE

In this section we describe the experimental sequence (Supplementary Fig. 2) and the implementation of the presented quantum circuits using our native gates in more detail. The NV electronic spin is chosen as the auxiliary qubit: first, it can be initialized and non-destructively read out by optical means; second, it can be used to directly implement two qubit gates with all of the ^{13}C nuclear-spin qubits as well as the ^{14}N nuclear-spin qubit. We use the ^{14}N nuclear spin as the flag qubit, and five ^{13}C nuclear spins as the data qubits.

Supplementary Fig. 2 provides an overview of the typical blocks in the experimental sequences. The qubit initialization and procedure is described in more detail in Supplementary Fig. 6, the encoding of the logical qubit and the layout of the data qubit echo sequences is given in Supplementary Fig. 8, the data qubit readout is given in Supplementary Fig. 6. Below we describe the native two-qubit gates in the system, the translation and compilation of the main text circuits into the experimental gate sequences and the implementation of echo sequences to decouple the qubits from each other and from the environment.

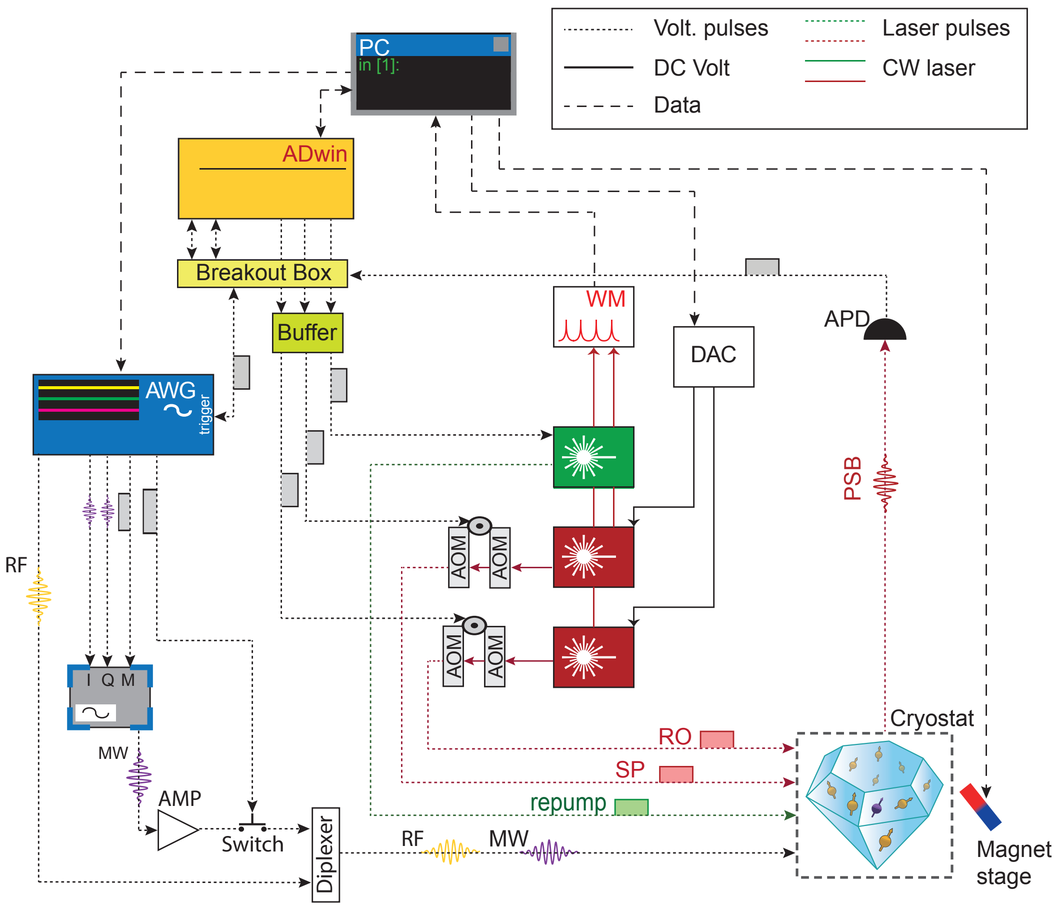

Supplementary Figure 1. Schematic of the experimental setup. **Control electronics**: We use a PC to program the real-time control cycle onto a micro-controller (Jaeger ADwin Pro II) and to program the waveforms with nanosecond resolution onto an arbitrary waveform generator (Tektronix AWG 5014c, 4.5V pk-pk). The communication between the different components of the setup is mainly done by the ADwin micro-controller, including triggering the AWG to start the pulse sequence and all real-time logic based on the measurement outcomes. **Lasers and Optics**: We use a green laser (515 nm, Cobolt MLD, on/off ratio of >135 dB) for charge state control and two resonant lasers (637 nm, Toptica DL Pro and New Focus TLB-6704-P) for initialization and readout. To generate optical pulses, the green laser can be directly modulated by the ADwin. For the two resonant lasers, we use acousto-optic modulators (AOMs) that are controlled by the ADwin micro-controller (through a buffer stage). To suppress the optical background noise we cascade two AOMs for each of the lasers (Gooch and Housego Fibre Q, total on/off ratio >100 dB). The frequencies of the two resonant lasers are measured using a wavemeter (HF-ANGSTROM WS/U-10U) and stabilized using a PC-controlled feedback loop (PID loop) to 2 MHz accuracy. We use a home-built confocal microscope to focus light onto the sample and to collect the emission. The microscope objective (Olympus MPLFLN 100x) is held in vacuum, at ambient temperature and mounted on XYZ piezo scanner (PI Q545) that is used to make 3-dimensional scans and to precisely align the NV center to the optical path. The XYZ piezo scanner is controlled via the ADwin. NV phonon-sideband (PSB) emission is separated from reflected laser light using a long-pass filter (640 nm) and collected on an avalanche photodiode (APD, Laser Components). The APD signal is sent to the ADwin via a breakout box that includes a pulse stretcher. Detected photons are counted with the ADwin counting module. **Microwave and RF**: Microwave pulses are generated using a vector source (SGS100). The frequencies, timings, shapes and phases of the pulses are controlled via the AWG through IQ- and pulse-modulation. Single-sideband modulation at 250 MHz is used to spectrally isolate the control pulses from low frequency noise of the AWG, which is filtered using a 175MHz high-pass filter. The MW signal is amplified by a microwave amplifier (AR 25S1G6). We use a fast microwave switch (TriQuint TGS2355-SM, suppression ratio of 40 dB, controlled by the AWG in combination with a home-built driver) to protect the NV from the amplifier noise while idling. Video leakage noise generated by the switch is filtered with a high pass filter. The RF pulses for nuclear spin control are directly synthesized by the AWG. The MW and RF signals are combined using a diplexer and the output is fed to the diamond sample. **External magnetic field**: We apply a static magnetic field, $B_z \approx 403$ G, along the NV-axis using a permanent room-temperature neodymium magnet which is mounted on an XYZ translation stage to control the strength and the direction of the magnetic field. We stabilize the magnetic field strength to < 3 mG and the magnet is aligned to the NV-axis with an uncertainty of 0.07° using thermal echo sequences (see Ref. [3] for details of magnetic field stability and the alignment procedure). **Cryogenics**: The sample is held in a closed cycle cryostat (Montana Cryostation) at a temperature of 3.7 K.

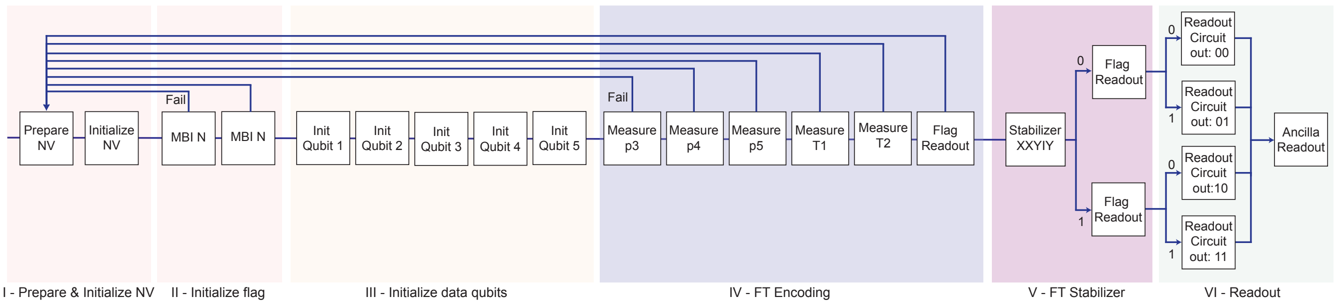

Supplementary Figure 2. Block diagram of the experimental sequence and logic. **I.** The NV center is prepared in the negative charge state and on resonance with the lasers, and then initialized into the $m_s = 0$ state through optical spin pumping [5]. See Supplementary Fig. 3 for details. **II.** The ^{14}N nuclear-spin flag qubit is initialized using measurement-based initialization (MBI), which is performed twice to improve the initialization fidelity (Supplementary Fig. 6). **III.** The five data qubits are initialized sequentially by SWAP sequences that transfer the electron spin state ($|0\rangle$) onto the data qubits (Supplementary Fig. 6). **IV.** To fault-tolerantly encode the logical qubit, we measure the logical operators p_3, p_4, p_5, the verification operators $T_1 = p_2 p_4 p_5$, $T_2 = p_1 p_3 p_5$, and the flag qubit. **V.** After a successful logical state preparation, we measure the stabilizer $s_1 = XXYIY$ with a flag qubit measurement check. **VI.** Finally, to characterize the post measurement state and obtain the logical state fidelity, we perform multi-qubit readout of the data qubits by mapping the required correlation to the auxiliary qubit through a series of single- and two-qubit gates (Supplementary Fig. 6), which can then be optically read-out. The branches and return arrows in the diagram indicate real-time decisions and feedforward based on the measurement outcomes. Optional extra blocks for logical gates (Fig. 4 in the main text) are not shown.

A. NV preparation and initialization

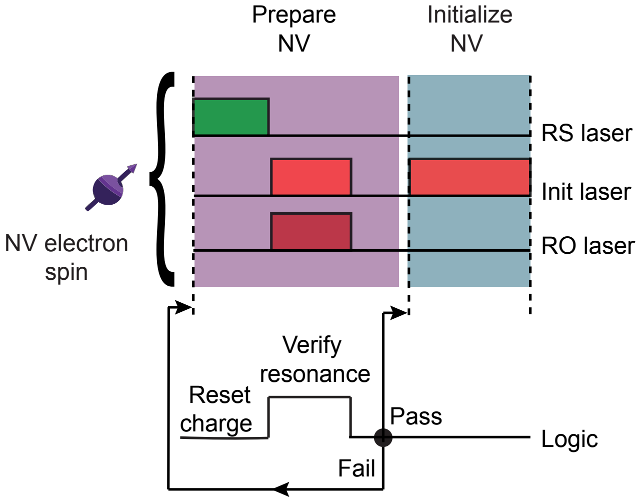

Supplementary Figure 3. NV preparation and initialization. **NV preparation**: The NV centre is prepared in the negative charge state and brought on resonance with the lasers used for the initialization (Init laser) and readout (RO laser) steps. We simultaneously apply the Init and RO lasers for 150 µs and count the number of detected photons (wavelength ∼ 637 nm, RO laser resonant with $m_s = 0 \leftrightarrow E_x$ transition and Init laser resonant with $m_s = \pm 1 \leftrightarrow E_{1,2}$ transition) [5]. If the number of detected photons exceeds a certain threshold, the NV is in the negative charge state and on resonance with both lasers, and the sequence proceeds to the next step. If not, the charge reset laser (RS, wavelength ∼ 515 nm) is applied for 1 ms and the same process is repeated until success [5]. **NV initialization**: the NV electron spin is initialized into the $m_s = 0$ state through spin pumping on the $E_{1,2}$ transition (Init laser, 100 µs) [5].

B. Two-qubit gates

Our native two-qubit gates are performed by applying dynamical decoupling sequences on the NV electron spin, in resonance with a targeted nuclear spin [6, 7]. This leads to controlled rotations which are equivalent to the standard CNOT gate up to single qubit rotations. See Supplementary Fig. 4 for the gate definitions.

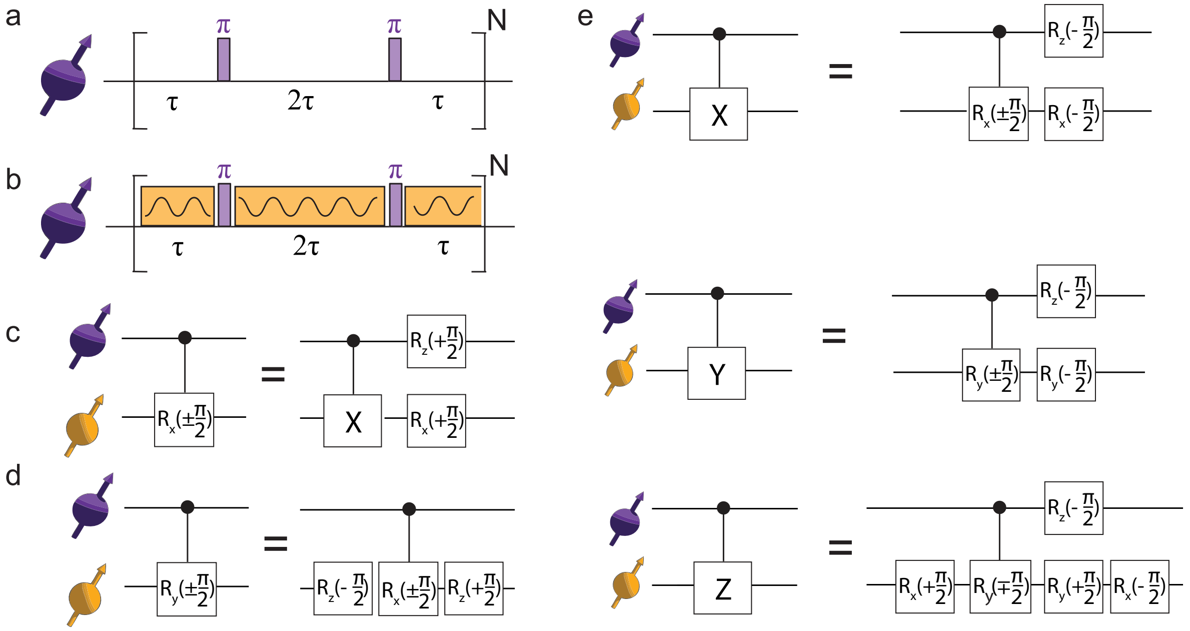

Supplementary Figure 4. Experimental two-qubit gates. a) Sequence for a DD gate: A decoherence-protected electron-nuclear gate is realized by a dynamical decoupling sequence of N equally spaced π-pulses on the electron spin of the form $(\tau_r - \pi - \tau_r)^N$, and setting τ to be resonant with one of the ^{13}C nuclear spins [6, 7]. This design relies on the presence of a significant hyperfine-interaction component perpendicular to the applied magnetic field [6]. b) Sequence for a DDRF gate: if the perpendicular hyperfine coupling is small, we perform two-qubit gates by interleaving the dynamical decoupling sequence with resonant radio-frequency pulses [7]. c) Both sequences realize an electron-controlled nuclear gate, $CR_x(\pm\pi/2)$, which is equivalent to a standard CNOT gate up to single qubit rotations. Note that $CR_x(\pm\pi/2) = |0\rangle\langle 0| \otimes R_x(+\pi/2) + |1\rangle\langle 1| \otimes R_x(-\pi/2)$. d) Applying two-qubit gates around arbitrary axes is done by applying phase-shift gates. For example, $CR_y(\pm\pi/2)$ is implemented using $CR_x(\pm\pi/2)$ by adding single-qubit phase-shift gates as shown here. e) Implementation of the standard controlled gates using our two-qubit gate schemes and extra single qubit rotations.

C. Phase synchronization and pulse timing

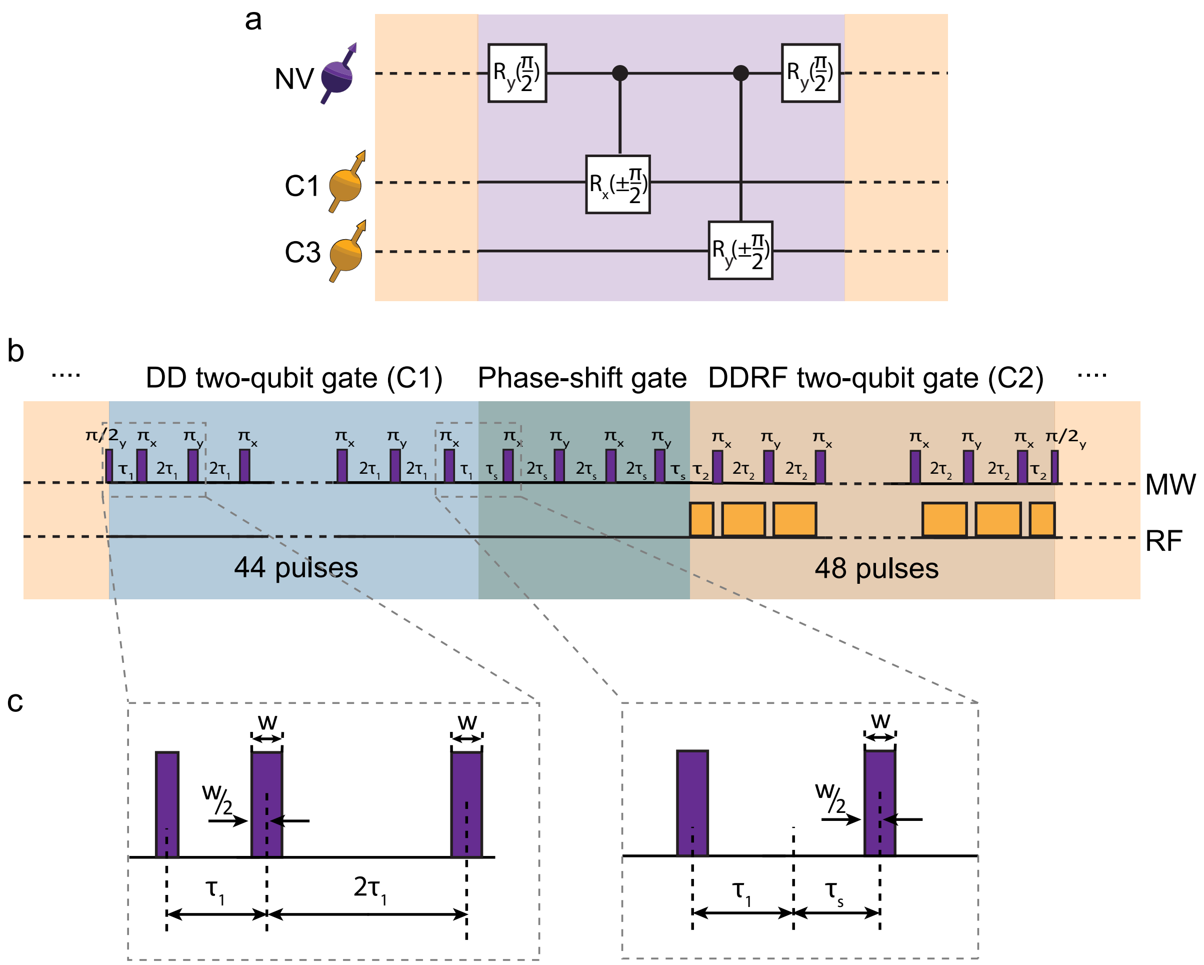

Supplementary Figure 5. Example gate sequence illustrating timing of the pulses and phase synchronization of the qubits. a) The target quantum circuit. b) The underlying pulse sequence to implement the circuit in (a). The single-qubit electron rotations are performed by applying microwave pulses. We use Hermite pulse envelopes [4, 8] to obtain effective MW pulses without initialization of the intrinsic ^{14}N nuclear spin (square pulse shapes are shown in the figure for simplicity). Decoherence-protected electron-nuclear two-qubit gates are realized by a dynamical decoupling sequence as explained in Supplementary Fig. 4. To mitigate pulse errors, we alternate the phases of the pulses following the XY-8 scheme [4]. Our native two-qubit gates rotate each nuclear spin along a fixed rotation axis in the lab frame, which is set by its hyperfine interaction (for DD gates) or the RF waveform (for DDRF gates). Applying two-qubit gates around arbitrary axes is done by applying phase-shift gates. To this end, the phases of the nuclear spin qubits are tracked throughout the pulse sequences and phase-shift gates are used to achieve the required phase on the target qubit before applying a two-qubit gate (see pseudocode 3 in section VIII for details). If the NV electron spin is in a superposition state, the phase-shift gates are implemented using a 4-pulse dynamical decoupling sequences (XY-4 scheme) to mitigate electron spin decoherence; otherwise they are implemented using free evolution wait times (see pseudocode 3 for details). A similar approach is used when applying RF pulses to the nuclear spins for single-qubit gates, as the AWG-synthesized pulses have set phases with respect to a fixed lab frame. c) Zoom-in showing how the pulses are timed. As the electron MW pulses have finite durations, the pulse spacing is determined from the pulse centers.

D. Initialization and final readout of the data qubits

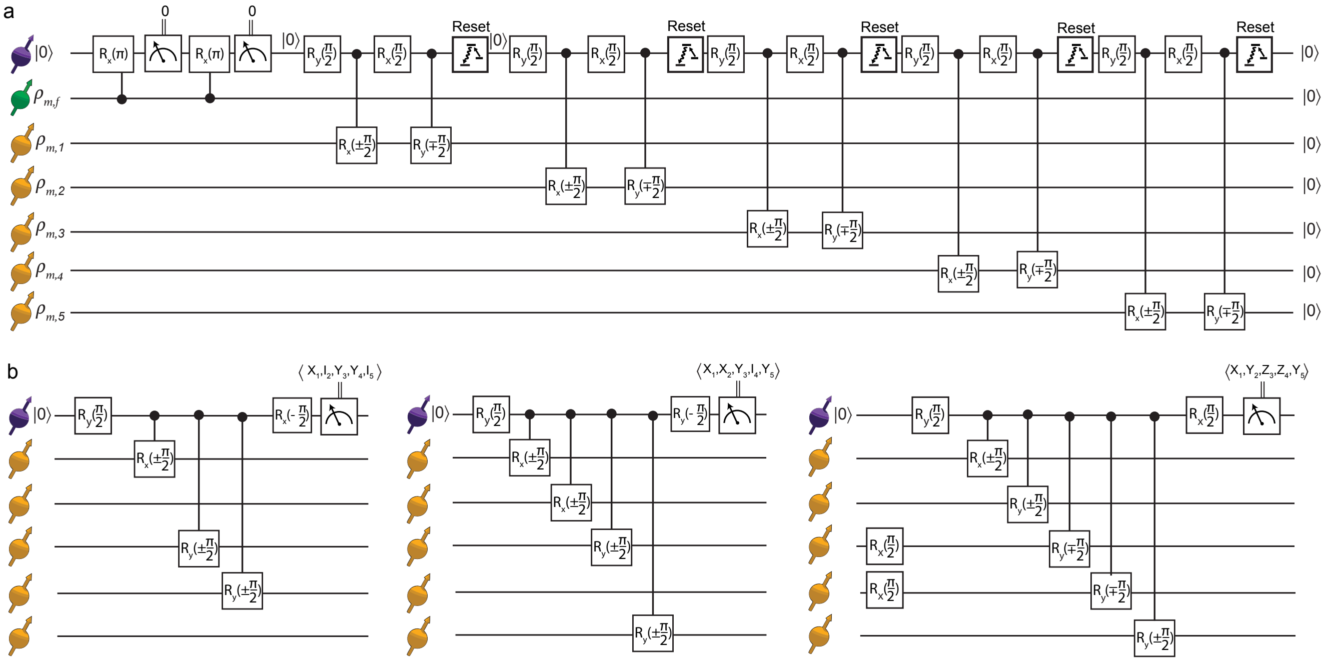

Supplementary Figure 6. Initialization and readout of the data qubits. a) Initialization of the nuclear-spin qubits. First, the ^{14}N nuclear-spin flag qubit is initialized using a measurement-based initialization sequence (performed twice to improve the initialization fidelity) [7]. Afterwards, the five data qubits are initialized sequentially by SWAP sequences that transfer the electron spin state ($|0\rangle$) onto the data qubits. An optical pulse is used to reset the electron state to $|0\rangle$ following each SWAP step. b) Measuring multi-qubit operators of the data qubits is performed by mapping the required correlation to the auxiliary qubit (through controlled qubit rotations) and then reading out the auxiliary qubit. As each additional electron-controlled nuclear gate adds a $\pi/2$ phase shift to the electron spin (Supplementary Fig. 4), the final phase of the $\pi/2$ readout pulse accounts for the number of the spins being measured (i.e., the phase depends on the number of non-identity terms in the measured operator). Three examples are shown that capture the main idea. Such measurements are used both to realize non-destructive stabilizer measurements, as well as to tomographically measure the data qubits to characterize the final state.

E. Cross-phase calibrations

While applying the DD or DDRF two-qubit gates (targeting a given nuclear-spin qubit), the other qubits pick up a systematic phase shift [6]. In experiments, we typically calibrate and correct for this effect. Supplementary Fig. 7 shows the calibration procedure. The same procedure is also used to calibrate for cross-phases while applying single-qubit RF gates on the nuclear spins.

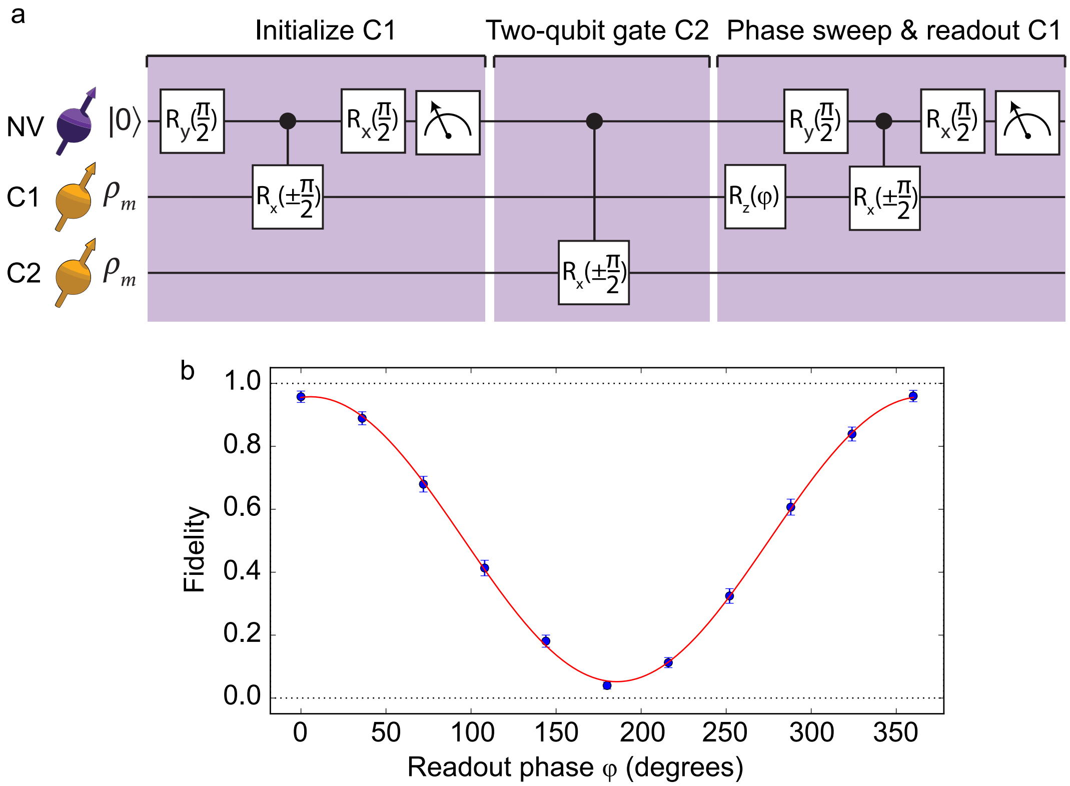

Supplementary Figure 7. Cross-phase calibrations for the two-qubit gates. a) Calibration sequence to measure the systematic phase shift that non-target qubits acquire while applying an electron-nuclear two-qubit gate. First, the non-target spin is initialized by measurement (see Ref. [1] for details). Afterwards, the DD or DDRF two-qubit gate is applied on the target nuclear-spin qubit. Finally, the non-target qubit is measured while varying the readout phase ϕ (via the $R_z(\phi)$ gate). The phase shift (ϕ_c) is then obtained from fitting the measured signal to the function $S = a + A \cdot \cos(\phi + \phi_c)$, where a, A, and ϕ_c are the fit parameters. b) Example calibration signal: here C1 is the initialized spin and C2 is the target spin. The obtained phase correction is $\phi_c = 5(1)$ degrees.

F. Circuit compilation

In order to implement the circuits presented in the main text, we first translate all gates into our native gates (Supplementary Fig. 4) and afterwards compile the circuit to reduce the total number of single-qubit gates. Supplementary Fig. 8 shows the compiled circuit diagram for the encoding circuit presented in Fig. 3a in the main text. Note that the same approach is also taken when for performing stabilizer measurements on the logically encoded state (Fig. 5a in the main text). Supplementary Fig. 9 shows the entire compiled circuit diagram in our native gate scheme including data and flag qubits initialization, FT encoding, FT stabilizer measurement, echo stages, and an example for final data qubit readout.

Measurement	Success probability
p_3	0.39
p_4	0.41
p_5	0.41
T_1	0.62
T_2	0.49
Flag	0.76

Supplementary Table 1. Success probabilities for the stabilizer measurements in the encoding scheme (i.e., probabilities to obtain +1 eigenvalues). The average success probability for the FT encoding scheme is 0.015 (multiplication of the individual success probabilities).

G. Echo sequences for the data qubits

An important challenge is to mitigate decoherence of the data qubits in order to leave few errors to be dealt with by error correction. The used circuits involve up to 40 two-qubit gates (including those required for the initialization and readout of the data qubits); the typical gate time is 0.5 to 1 ms (Supplementary Table 9). This makes the total sequence time much longer than the dephasing time of the individual nuclear spins ($T_2^* = 3 - 17$ ms for the data qubits, see Supplementary Table. 3). An effective way to overcome this dephasing is to use spin echoes, which can increase the coherence times to several seconds [7]. The designed echo sequence should minimize any idle waiting time during which the electron spin is in a superposition state (i.e., not in an spin eigenstate). We found that using two echo stages provides a general solution such that the nuclear spins can refocus at the required points (see the supplementary material of Ref. [7] for details).

An additional challenge here is that the small couplings between the nuclear-spin data qubits become non-negligible for the long sequences implemented here (up to 100 ms when including the echo pulses) [3]. From the measured coupling strengths (Supplementary Table 5), we find that the most relevant ones are those between data qubits C3$\leftrightarrow$C2 and C3$\leftrightarrow$C5 (16.90(4) Hz and 12.96(4) Hz respectively). To overcome this we use asynchronous echo sequences, i.e., we do not echo all the spins simultaneously. By applying a single echo pulse on qubit C3, it is also decoupled from the other 4 qubits, for which we apply two echo pulses (see Supplementary Fig. 8 for illustration). We adopt this approach for all echo stages used in this work. More complex sequences that can decouple all nuclear-nuclear couplings are possible, but in the current system the available Rabi frequencies pose a limitation. Such improved decoupling sequences are anticipated to be feasible with future experimental upgrades to enable faster RF pulses (higher RF power using low noise amplifiers, RF switches, and a better RF delivery to the sample (e.g. RF coils) to avoid sample heating).

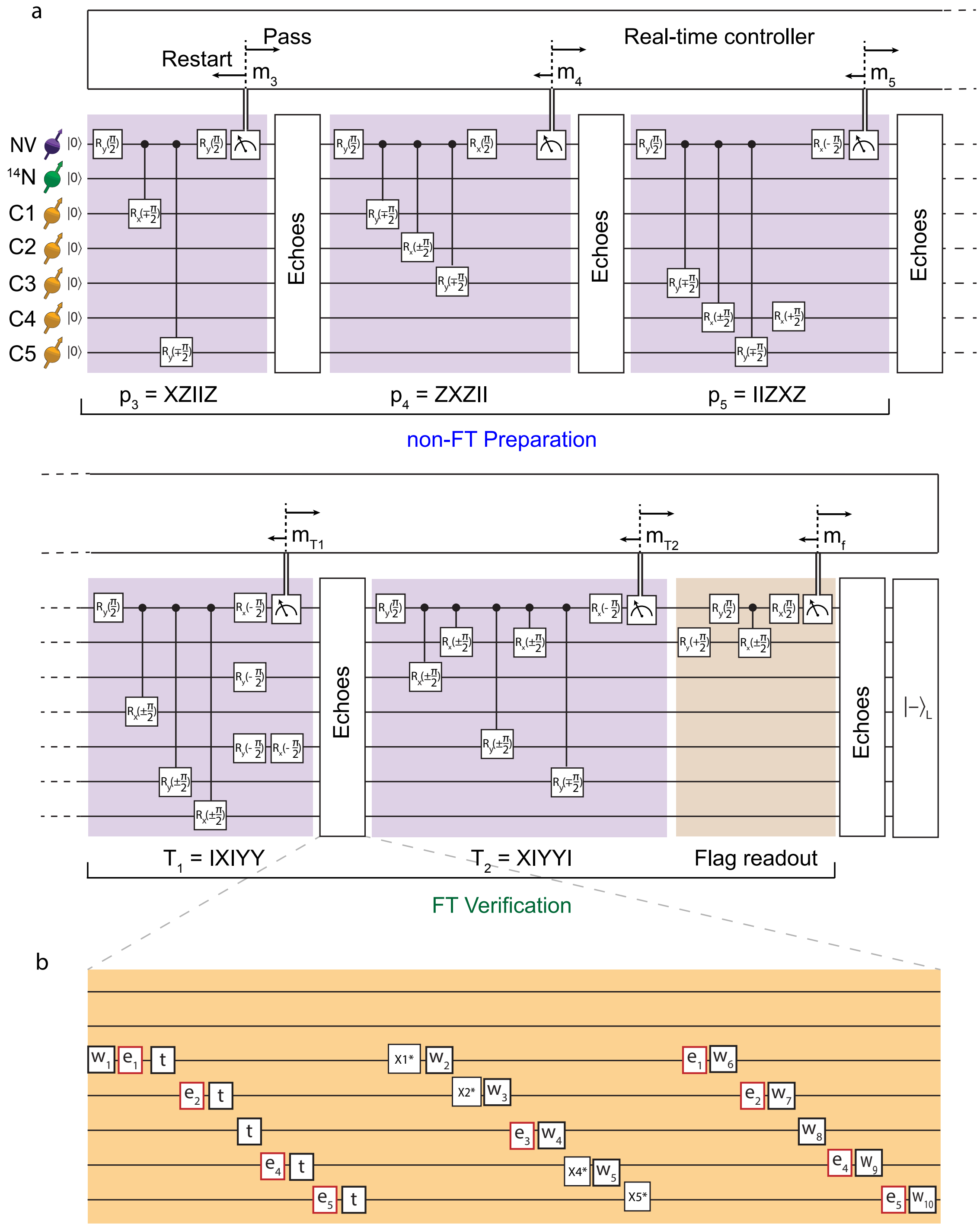

Supplementary Figure 8. Experimental implementation of the logical state encoding scheme. a) The circuit diagram in Fig. 3a in the main text is converted to our native gates and then compiled to minimize the number of single qubit gates. The ^{13}C nuclear spins are first initialized into $|00000\rangle$ (Supplementary Fig. 6). Note that the compiled circuit prepares the target state up to single qubit rotations ($U_{FT} = R_z(\pi) \otimes R_x(\pi) \otimes R_x(\pi)R_y(\pi/2) \otimes R_x(\pi)) \otimes R_z(\pi/2)$) which are normally compiled with the next step (e.g. data qubit readout, a stabilizer measurement, or an application of a logical gate). We numerically simulate the compiled circuit (using the QuTip Python toolbox [9]) and compare the obtained output state (up to the single qubit rotations U_{FT}) to the standard circuit diagram to verify the performance. b) We apply echo stages on the nuclear spins between the stabilizer measurements to mitigate the nuclear-spin decoherence and unwanted couplings. The echos are applied in asynchronous form: for qubit C3 only a single echo pulse is applied per stage, so that it is also decoupled from the other 4 qubits on which we apply two echo pulses per stage. This simultaneously enhances the nuclear-spin coherence time and avoids effects of nuclear-nuclear interactions between the nuclear-spin qubits. Boxes $e_1, e_2, ..., e_5$ correspond to spin echo pulses on those spins, with unique lengths calibrated to maximize the pulse fidelity. Boxes $X_1^*, .., X_5^*$ are rephasing points, and at these points single-qubit rotations can be applied (in case of a required basis transformation). Boxes $W_1, W_2, ..., W_{10}$ and t are unique waiting duration times derived from a set of simultaneous equations following the concepts presented in Ref. [7].

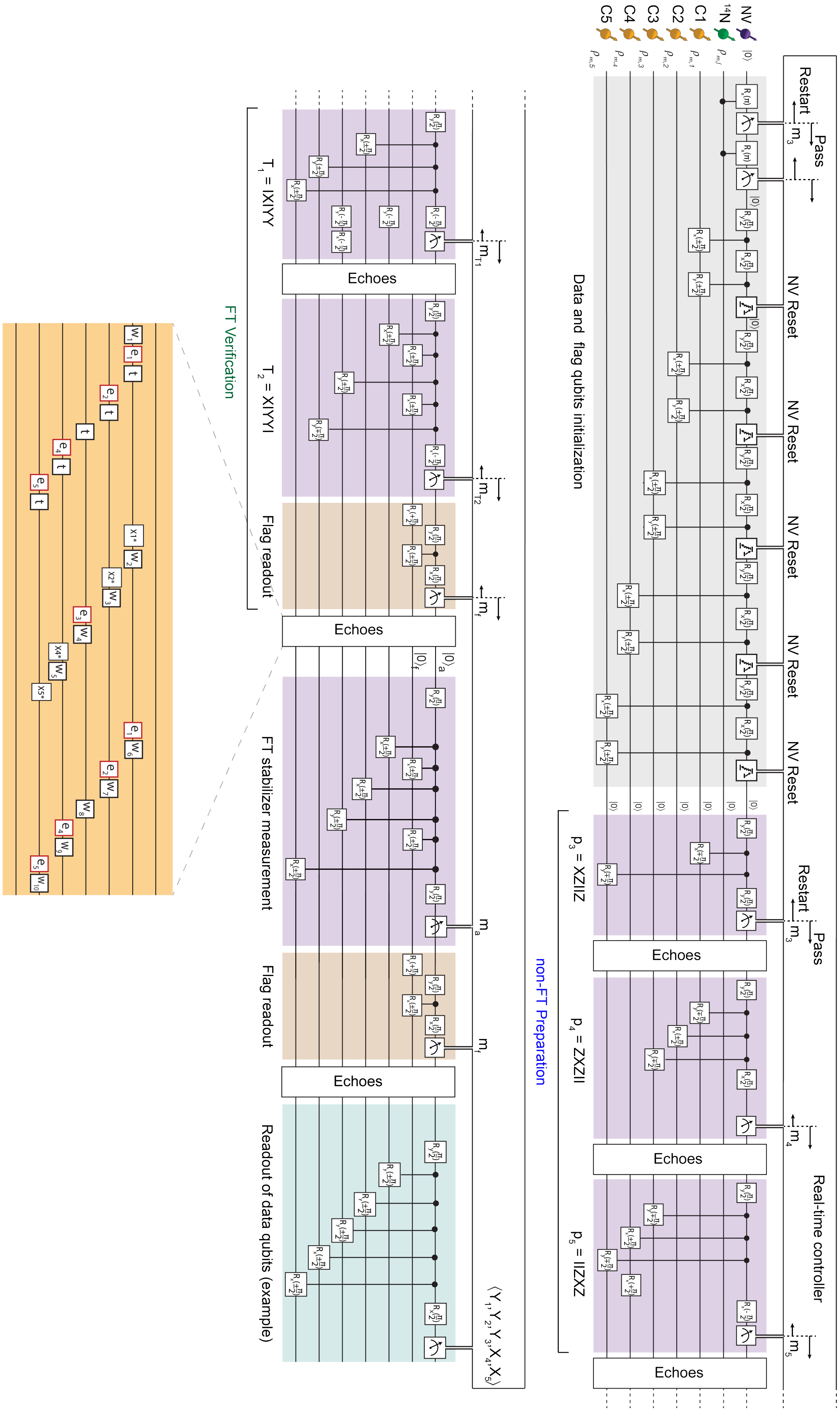

Supplementary Figure 9. The entire circuit diagram in our native gate scheme including data and flag qubit initialization, FT encoding, FT stabilizer measurement, echo stages, and an example for final data qubit readout.

IV. PARAMETERS OF THE NUCLEAR-SPIN QUBITS

	$\omega_0/2\pi$ [Hz]	$\omega_{-1}/2\pi$ [Hz]	$\omega_{+1}/2\pi$ [Hz]	$A_\parallel/2\pi$ [kHz]	$A_\perp/2\pi$ [kHz]
C1	431956(1)	469020(1)	396542(1)	-36.308(1)	26.62(4)
C2	431962(1)	408317(1)	457035(1)	24.399(1)	24.81(4)
C3	431961(1)	218828(1)	645123(1)	213.154(1)	3.0(4)
C4	431958(1)	413477(1)	454427(1)	20.569(1)	41.51(3)
C5	431962(1)	480625(1)	383480(40)	-48.58(2)	9(2)
^{14}N	5069110(1)	2884865(1)	7263440(1)	2189.288(1)	-

Supplementary Table 2. **Precession frequencies and hyperfine couplings for the nuclear spin qubits.** ω_0, ω_{-1}, and ω_{+1} are the measured nuclear precession frequencies for the $m_s = 0, -1$ and $+1$ electron spin projections respectively, obtained from least-squares fits of Ramsey signals. $A_\parallel$ and $A_\perp$ are the hyperfine interaction components parallel and perpendicular to the applied magnetic field. For the ^{14}N spin the frequencies given are for the $m_I = 0 \leftrightarrow m_I = -1$ transition, and the parallel hyperfine component is taken as $(\omega_{+1} - \omega_{-1})/2$.

	T_2^* ($m_s = -1$) [ms]	T_2^* ($m_s = 0$) [ms]	T_2 ($m_s = -1$) [s]	$T_2^{\alpha=256}$ ($m_s = -1$) [s]
C1	9.2(8)	9.1(5)	0.53(6)	6.8(8)
C2	15.6(8)	17.2(4)	0.62(3)	12.9(4)
C3	12.0(6)	10.0(3)	0.77(4)	25(4)
C4	11.9(5)	12.3(3)	0.68(3)	7.4(8)
C5	3.7(2)	3.6(2)	0.59(2)	13(2)
^{14}N	23.2(7)	25.1(7)	2.3(2)	63(2)

Supplementary Table 3. **Coherence times for the nuclear-spin qubits [7].** T_2^* is the dephasing time obtained from a least-squares fit of Ramsey signals, with evolution for the $m_s = -1$ and $m_s = 0$ electron spin projections. T_2 is the coherence time obtained from a least-squares fit of a spin echo experiment, with free evolution measured for the $m_s = -1$ electron spin projection. $T_2^{\alpha=256}$ is the coherence time obtained from a least-squares fit of a dynamical decoupling experiment with $\alpha = 256$ pulses, with the electron spin in the $m_s = -1$ spin projection.

	N	τ (µs)	Gate type	Gate duration (µs)	F_{gate}	F_{init}	RF π-pulse duration (µs)
C1	44	7.218	DD	635	0.99(1)	0.985(5)	1096
C2	32	6.540	DD	419	0.99(1)	0.980(5)	1606
C3	48	16.204	DDRF	1556	0.97(1)	0.965(5)	1330
C4	22	11.250	DD	495	0.97(1)	0.970(5)	929
C5	90	4.932	DD	888	0.95(1)	0.985(5)	1173
^{14}N	12	16.204	DDRF	389	-	0.997(11)	278

Supplementary Table 4. **Gate parameters and fidelities for the nuclear spin qubits.** N and τ are the parameters used for the dynamical decoupling sequence to implement electron-nuclear two-qubit gates (Supplementary Figure 3), where N is the total number of electron π-pulses, and 2τ is the interpulse delay. Gate type refers to whether the dynamical decoupling sequence is interleaved with RF pulses (DDRF) or not (DD), see Supplementary Fig. 4. F_{init} is the measured initialization fidelity for the nuclear-spin qubits [7]. F_{gate} is the gate fidelity estimated from the electron-nuclear Bell-state fidelity [7]. For the ^{14}N flag qubit, Bell-state tomography is challenging because the optical read-out of the auxiliary qubit induces rapid dephasing. A lower bound of 0.94(1) is extracted from such measurements, but the fidelity is likely much higher.

	C1	C2	C2	C4	C5
C1	-	2.64(4)	2.8(1)	2.2(4)	2.6(1)
C2	2.64(4)	-	16.90(4)	1.08	1.42
C3	2.8(1)	16.90(4)	-	3.12(8)	12.96(4)
C4	2.2(4)	1.08	3.12(8)	-	4.0(1)
C5	2.6(1)	1.42	12.96(4)	4.0(1)	-

Supplementary Table 5. **Qubit-qubit coupling.** Measured coupling strength (C_{ZZ}) in Hz between the nuclear-spin data qubits. Values without uncertainties are calculated from the nuclear spin coordinates, rather than measured directly [3].

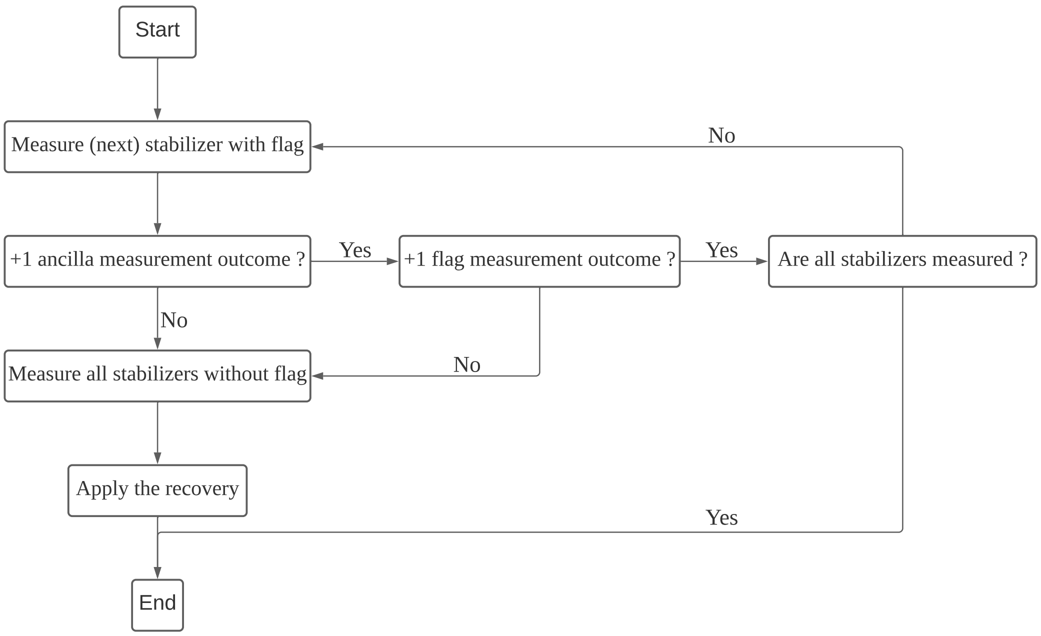

Supplementary Figure 10. The flowchart of a full flag error correction cycle proposed by Chao and Reichardt [10]. For the 5-qubit code, the stabilizers are $s_1 = XXYIY, s_2 = YXXYI, s_3 = IYXXY$ and $s_4 = YIYXX$. The stabilizer measurements without flag give a 4-bit error syndrome, which determines the recovery operation. Note that the error syndrome is interpreted differently depending on whether there is a raised flag. In the experiments presented in this work, we apply one stabilizer measurement of this error correction cycle and characterize the resulting post-measurement state.

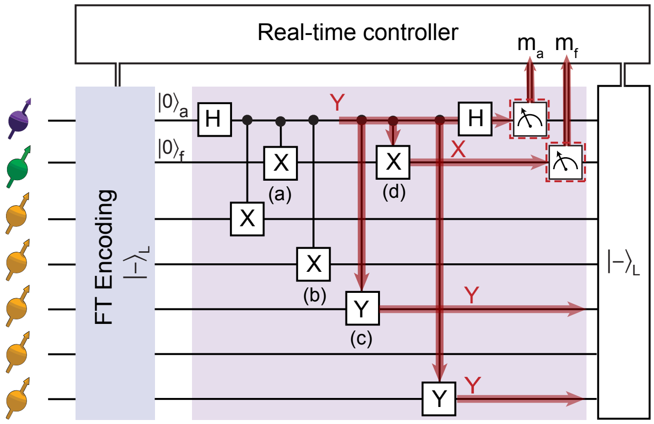

Supplementary Figure 11. The circuit for measuring the stabilizer $s_1 = XXYIY$ with flag on the encoded state. Given this specific order of gates, the Y error on the auxiliary qubit is a malicious fault for the logical state $|-\rangle_L$, as it will propagate to the data qubits to become $Y_3X_5 = -Z_4 \cdot \overline{Z} \cdot s_1 \cdot s_3 \cdot s_4$. This 2-qubit error Y_3Y_5 will lead to a logical Z error after a round of noiseless error correction. However, this Y error will also propagate to the flag auxiliary qubit and flip its measurement outcome, which provides additional information: a flipped flag measurement heralds the possibility of such a malicious fault.

V. FLAG FAULT-TOLERANT QUANTUM ERROR CORRECTION

In our experiment we realize fault-tolerant logical state preparation and flagged stabilizer measurements of a distance-3 5-qubit quantum error correction code. Here we first briefly review the idea of (flag) fault tolerance and distance-3 codes [10, 11]. Then we analyze the error syndromes in the context of the experimentally applied stabilizer measurement (Supplementary Fig. 11). In section VI we then provide a proof of the fault-tolerance of our

gate (b): fault	data qubit error	gate (c): fault	data qubit error
XI (YI)	Y_3Y_5	XI (YI)	Y_5
XX (YX)	$X_2Y_3Y_5 \equiv X_1$	XX (YX)	X_3Y_5
XY (YY)	$Y_2Y_3Y_5 \equiv X_1Z_2$	XY (YY)	Y_3Y_5
XZ (YZ)	$Z_2Y_3Y_5 \equiv X_1Y_2$	XZ (YZ)	Z_3Y_5
gate (a): fault	data qubit error	gate (d): fault	data qubit error
XZ (YZ)	$X_2Y_3Y_5 \equiv X_1$	XX (XY)	Y_5
XI (YI)	$X_2Y_3Y_5 \equiv X_1$	YX (YY)	Y_5
IX (IY)	I	IX (IY)	I
ZX (ZY)	I	ZX (ZY)	I

Supplementary Table 6. Single faults that can flip the flag qubit measurement in the stabilizer $s_1 = XXYIY$ measurement circuit (Supplementary Fig. 11). Such a fault is either induced by a faulty two-qubit gate or a single-qubit idling error after the gate. A readout error of the flag qubit can lead a different correction but does not induce an error on the output state directly. These 2-qubit Pauli errors are arranged in the order of control and target qubit respectively and they result in certain data qubit errors on the output state (errors in parentheses lead to the same data qubit errors). Syndromes of these resulting data qubit errors remain distinguishable, hence a perfect round of stabilizer measurements would identify and correct them (Supplementary Table 7). Note that here we assume only one fault during the circuit of measuring s_1 and the state preparation is noiseless.

heralded preparation protocol (Fig. 3a in the main text).

A distance-3 code can correct an arbitrary single-qubit error. This ability is, however, based on the assumption of realizing syndrome extraction by ideal stabilizer measurements. In practice, one has to design fault-tolerant error syndrome extraction protocols which work when the operations involved are noisy. For distance-3 codes, a unit of fault-tolerant quantum error correction or syndrome extraction satisfies the following criteria [11, 12]: (1) If the logical input state has a single qubit error and the syndrome extraction unit is noise-free, the output state is the logical input state with its error removed; (2) If the logical input state is noise-free and there is a single fault in the syndrome extraction unit, the output state has at most a single-qubit error on top of the given input state; (3) If the input state is an arbitrary state, and there is at most one single fault in the syndrome extraction unit, then the output state is any encoded state with at most a single-qubit error. For the 5-qubit code, the last requirement is automatically fulfilled. For details about fault-tolerance, we refer the reader to Refs. [11, 12].

Note that a single fault constitutes the insertion of any Pauli error at a location in a circuit, where locations can be: a qubit idling step, a qubit measurement, a qubit preparation, a single or two-qubit gate. In case the location is a two-qubit gate, a single fault is the insertion of any of the 15 two-qubit Pauli errors after the action of the gate. For a single-qubit measurement a fault is getting the wrong measurement outcome.

The basic idea of flag fault-tolerance is to use the additional information provided by the flag qubit measurements to correct certain two-qubit errors [10, 11, 13]. For example, we consider the measurement of $s_1 = XXYIY$ using the circuit in Supplementary Fig. 11. We assume that the logical preparation is noise-free and there is a single fault in measuring s_1. It can then be shown that: (1) if the flag qubit is not flipped, there is at most a single-qubit error on the output state; (2) otherwise, errors on the data qubits can only be X_1, X_1Z_2, X_3Y_5, Y_3Y_5, Z_3Y_5, X_1Y_2 and Y_5, see Supplementary Tab 6. If we were to apply a round of noiseless error correction on the output state, these errors can be identified and corrected as they have distinguishable syndromes. Each syndrome uniquely points to what error has happened (Supplementary Table 7).

To implement a complete flag error correction cycle, one repeatedly measures stabilizers (with and without flag) in a conditional form, until the error on the logical qubit can be unambiguously identified [10, 11]. We summarize the protocol proposed by Chao and Reichardt [10] as a flow chart in Supplementary Fig. 10. One can find a similar version of flag FT error correction protocol in Ref. [11]. Such flag error correction protocols can also be used to deterministically prepare a logical state that is an eigenstate of a logical Pauli operator. This is realized by performing FT error correction with respect to an extended stabilizer set, where the logical Pauli operator is treated as a stabilizer [11].

FT preparation of logical states using the typical non-fault tolerant method to prepare graph states and then applying a conditional verification step is also possible, as originally proposed by Chao and Reichardt in ref. [10]. However, this preparation scheme requires directly applying many controlled-phase gates between the data qubits which are not native to our platform. In this work, we develop a simpler heralded FT preparation scheme (Fig. 3a in the main text). Our scheme uses non-destructive parity measurements, and does not require direct two-qubit gates between the data qubits. Note that the success rate of the heralded scheme generally improves with increasing gate fidelities.

A. Stabilizer measurement with flag

In the experiments we first prepare the logical state $|-\rangle_L$ and then implement the measurement of the stabilizer $s_1 = XXYIY$ using a flag qubit (Supplementary Fig. 11). This measurement is a primitive of the Chao and Reichardt protocol described above. To calculate the logical state fidelity, we imagine a round of perfect stabilizer measurements on the experimentally-obtained output state. This round of stabilizer measurements can use the flag information to apply optimal recoveries.

If the flag qubit measurement is flipped, all possible errors on the output state due to a single fault are listed in Supplementary Table 6. Note that this table is obtained with the assumption that the input state is a logical state without error and there is only one fault in measuring s_1. However, errors outside the table could occur due to an incoming error on the logical state or possibly multiple faults in the circuit of measuring s_1. In such cases, the strategy of the imagined noiseless error correction round is to simply apply the corresponding single-qubit recovery, as if the flag qubit is not flipped. Therefore, when the flipped flag qubit measurement is taken into account, the set of correctable errors is

$$\mathcal{E}' = \{I, X_1, X_3Y_5, Z_1, X_2, Y_2, Z_3Y_5, X_1Y_2, Y_3, Z_3, X_4, Y_4, Y_3Y_5, X_5, Y_5, X_1Z_2\}. \quad (S6)$$

The corresponding syndromes are listed in Supplementary Table 7.

s_1	s_2	s_3	s_4	$\mathcal{E}$	$\mathcal{E}'$
+1	+1	+1	+1	I	I
+1	-1	+1	-1	X_1	X_1
-1	-1	+1	-1	Z_1	Z_1
-1	+1	+1	+1	Y_1	X_3Y_5
+1	+1	-1	+1	X_2	X_2
-1	-1	-1	+1	Z_2	Z_3Y_5
-1	-1	+1	+1	Y_2	Y_2
-1	+1	+1	-1	X_3	X_1Y_2
-1	-1	-1	-1	Z_3	Z_3
+1	-1	-1	+1	Y_3	Y_3
+1	-1	+1	+1	X_4	X_4
+1	-1	-1	-1	Z_4	Y_3Y_5
+1	+1	-1	-1	Y_4	Y_4
-1	+1	-1	+1	X_5	X_5
+1	+1	+1	-1	Y_5	Y_5
-1	+1	-1	-1	Z_5	X_1Z_2

Supplementary Table 7. Each 4-bit syndrome (eigenvalues of the stabilizers $s_1 = XXYIY, s_2 = YXXYI, s_3 = IYXXY$ and $s_4 = YIYXX$) can correspond to different Pauli errors. When $s_1 = XXYIY$ is measured using the circuit in Supplementary Fig. 11 and the flag is raised, $\mathcal{E}'$ represents the set of correctable errors with the flag information taken into account. When the flag information is not taken into account, $\mathcal{E}$ is the set of correctable errors including all single-qubit Pauli errors.

VI. PROOF OF FAULT-TOLERANCE OF THE PREPARATION SCHEME

In this section we provide the theoretical proof that a single fault in the encoding circuit (Fig. 3a in the main text) leads to the correct preparation of $|-\rangle_L$ plus at most a single-qubit error, assuming that the conditions for accepting the state are fulfilled. The conditions are (1) the measurement outcomes of T_1 and T_2 are compatible with the measurement outcomes m_i of the logical operators p_i, i.e. $m_{T_1} = m_2 \times m_4 \times m_5$ and $m_{T_2} = m_1 \times m_3 \times m_5$; (2) the flag is not raised. Otherwise the state is rejected.

The modification of the preparation scheme with additional single-qubit gates does not change the fault-tolerance argument. Therefore, the same derivation applies to preparing other basis states by adding and then compiling transversal logical gates, as well as when we execute echo pulses or use different gate decompositions. In the experimental realization we additionally condition on the preparation runs which give $m_3 = +1, m_4 = +1, m_5 = +1$ since these measurement outcomes ($m_s = 0$ NV electron spin state) are more reliable. Such heralding does not affect the fault-tolerance arguments and we provide the more general proof here. In this section, we denote controlled-NOT and controlled-Y gates as CX and CY.

First, we note that for this 5-qubit code, any state $|-\rangle_L$ with more than 1 Pauli error is equivalent to either a $|+\rangle_L$ with at most 1 Pauli error or a $|-\rangle_L$ state with at most 1 Pauli error. This is due to the code being ‘perfect’: the

states $|\pm\rangle_L$ plus any single-qubit error are all orthogonal and there are $2 \times (1+15) = 2^5 = 32$ such states, spanning the full 5-qubit Hilbert space.

Hence we just need to prove that the preparation circuit does not lead to the state $|+\rangle_L$ with at most 1 Pauli error. For this it is useful to tabulate some incarnations of the logical Z and logical Y as these can bring $|-\rangle_L$ to $|+\rangle_L$ up to a global phase, see Supplementary Table 8.

$Z_L \equiv$	$Z_1Z_2Z_3Z_4Z_5$	$-Y_3Z_4Y_5$ (cyclic perm.)	$-X_2X_3Z_5$ (cyclic perm.)
$Y_L \equiv$	$Y_1Y_2Y_3Y_4Y_5$	$-Z_2Z_3Y_5$ (cyclic perm.)	$-X_1X_4Y_5$ (cyclic perm.)
$X_L \equiv$	$X_1X_2X_3X_4X_5$	$-X_2Y_4Y_5$ (cyclic perm.)	$-Z_1Z_4X_5$ (cyclic perm.)

Supplementary Table 8. Incarnations of the logical operators which can be obtained by multiplying the logical operators by stabilizers (weight-3 incarnations have minus signs). Any cyclic permutation (e.g., $-X_2X_3Z_5 \to -Z_1X_3X_4 \to -Z_2X_4X_5$) of a logical operator is an equivalent logical operator.

Throughout this proof, we assume Pauli frame corrections by keeping track of detected errors in classical logic. The classical information changes our interpretation of the final measurement outcome, which can be basically interpreted as applying noiseless recovery according to the measurement outcomes (Supplementary Table 9).

m_3	m_4	m_5	correction
+1	+1	+1	I
+1	+1	-1	Z_4
+1	-1	+1	Z_2
+1	-1	-1	X_3
-1	+1	+1	Z_1
-1	+1	-1	X_5
-1	-1	+1	Z_1Z_2
-1	-1	-1	Z_1X_3

Supplementary Table 9. Pauli corrections to bring the prepared state to $|-\rangle_L$ (Fig. 3a in the main text). The correction anti-commutes (resp. commutes) with all p_i with $m_i = -1$ (resp. $m_i = +1$) and commutes with p_1 and p_2. The corrections in the look-up table are not unique as one can apply a stabilizer or any incarnation of a logical X to them which leaves the state $|-\rangle_L$ unchanged (modulo overall phase).

Looking at the circuit in Fig. 3a in the main text, we can assume that the fault appears either in the non-FT preparation circuit and the verification circuit is fault-free, or vice versa. So we consider these cases separately as follows.

Case A: if the only fault occurs in the verification circuit

Since the only fault occurs in the verification circuit, the preparation circuit prepares the state $|-\rangle_L$ with no errors. We prove that the circuit is fault-tolerant by considering different cases:

- Single-qubit faults on data qubits (in idling or after gates) during the verification. These have the effect of either leading to a single-qubit error on the output state, or leading to a flipped measurement of T_1 or/and T_2 which does not satisfy the consistency check (in which case the output state is not accepted).
- A single measurement fault in the measurement of T_1, T_2 or the flag qubit measurement leads to the state not being accepted.
- A two-qubit fault after one of the two-qubit gates in T_1. If the action of this fault on the auxiliary qubit is Z or Y, then it flips the auxiliary qubit and the state is not accepted. Hence we assume the action on the auxiliary qubit is X. If the error $XP \equiv X \otimes P$, where P is some Pauli I, X, Y, Z acting on a data qubit, occurs after the first CX, it leads to outgoing error $P_2Y_4Y_5$ which is equivalent to $(X_2P_2)X_L$, hence a single-qubit outgoing error. If XP occurs after CY in the middle, it leads to outgoing error P_4Y_5: (1) Y_4Y_5 is equivalent to X_2X_L, (2) X_4Y_5 and Z_4Y_5 lead to the T_2 outcome being flipped and no acceptance, (3) $P = I$ corresponds to the single-qubit error Y_5. If XP occurs after the (last) CY, it leads to a single-qubit outgoing error P_5.
- A two-qubit fault after one of the two-qubit gates in T_2. If the action of this fault on the auxiliary qubit is Z or Y, then it flips the auxiliary qubit and the state is not accepted. Hence we assume that the action on the auxiliary qubit is X.

  Consider first the two-qubit gates between auxiliary and data qubits. If the fault XP occurs after the CX, then it does not flip the flag qubit, and leads to outgoing error $P_1Y_3Y_4$ which is equivalent to $(X_1P_1)X_L$, hence

inducing a single-qubit error. If the fault XP occurs after CY in the middle, it flips the flag qubit and the state is not accepted. If the fault XP occurs after the second CY, it leads to a single qubit error P_4.

Now consider CX gates between auxiliary and flag qubits. Note that an error on the flag qubit cannot propagate to the data qubits, we thus only consider a Pauli X error on the auxiliary qubit. An X error on auxiliary qubit after the first CX leads to Y_3Y_4, which is equivalent to X_1X_L. An X error on the auxiliary qubit after the second CX leads to the single-qubit error Y_4.

	p_1	p_2	p_3	p_4	p_5	$T_1 = p_2p_4p_5$	$T_2 = p_1p_3p_5$
Y_1		F	F	F			F
X_1		F		F			
Z_1			F				F
Y_2	F		F	F		F	
X_2	F		F				
Z_2				F		F	
Y_3	F			F	F		
X_3				F	F		F
Z_3	F						F
Y_4	F	F			F		
X_4	F	F				F	F
Z_4					F	F	F
Y_5		F	F		F		
X_5			F		F	F	
Z_5		F				F	

Supplementary Table 10. Effect of single qubit errors. F denotes that the eigenvalue is flipped upon application of the error, i.e., the error anti-commutes with the operator. For the logical state $|+\rangle_L$, both measurements of the verification checks T_1 and T_2 should give -1, which fails the verification. However, $|+\rangle_L$ with the errors highlighted in red (X_4 and Z_4) would pass the verification test as both verification checks are flipped.

Case B: if the only fault occurs in the preparation circuit

When the non-FT circuit gives the output $|-\rangle_L$ plus at most a single-qubit error, no matter whether this state passes the verification or not, the final output state can have at most a single-qubit error as the verification circuit is perfect. For output states $|+\rangle_L$ with at most a single-qubit error, we need to argue: (1) either they are caught by the verification circuit (2) or they cannot occur due to a single fault in the non-FT preparation circuit.

Note that the output $|+\rangle_L$ is a -1 eigenstate of p_1, p_2, p_3, p_4 and p_5. If the output state of the non-FT circuit is $|+\rangle_L$ with no error, both T_1 and T_2 should give measurement outcome -1 as they are products of three p_is. Such a state would be rejected by the verification circuit. Now we examine what possible single-qubit errors on $|+\rangle_L$ would lead to passing the verification test. We can see that these are the errors X_4 and Z_4 on top of $|+\rangle_L$, i.e., both anti-commute with the verification checks (Supplementary Table 10).

One can fully characterize these possible bad states $X_4\,|+\rangle_L$ and $Z_4\,|+\rangle_L$ by their syndromes $M_i = \pm 1$ (eigenvalues of $p_1, \ldots, p_5$ if we were to measure these logical operators noiselessly). Syndromes of the possible bad states (denoted by capital letters) are given as follows

$$X_4\,|+\rangle_L : M_3 = M_4 = M_5 = -1, \qquad M_1 = M_2 = +1, \tag{S7}$$

$$Z_4\,|+\rangle_L : M_1 = M_2 = M_3 = M_4 = -1, \qquad M_5 = +1 \tag{S8}$$

Now we argue that no single fault in the preparation circuit can lead to $X_4\,|+\rangle_L$ or $Z_4\,|+\rangle_L$. We look at various subcases:

1) If the only fault occurs before measuring p_4 and p_5. Then the Pauli corrections correctly fix the eigenvalues of p_4 and p_5 on the output so that $M_4 = M_5 = +1$. Both syndromes in Eq. (S7) and Eq. (S8) are therefore excluded.

2) If the only fault occurs in measuring p_4. Similarly, M_5 is fixed to be $+1$ due to correct Pauli corrections, so that the syndrome in Eq. (S7) is not possible. Then for the syndrome in Eq. (S8) we argue as follows:

- In the circuit of measuring p_4, only faults after CX in the middle can lead to non-trivial 2-qubit errors Z_2Z_3 and Y_2Z_3. These errors commute with $p_2 = ZIIZX$, hence M_2 is fixed to be $+1$. The syndrome in Eq. (S8) is therefore not possible.

- Up to a stabilizer or a logical X operator, other single faults can only induce single-qubit errors on the data qubits. Because any single-qubit error cannot anti-commute with $p_1 = IZXZI$, $p_2 = ZIIZX$ and $p_3 = XZIIZ$ at the same time, M_1, M_2 and M_3 cannot be -1 at the same time. The syndrome in Eq. (S8) is therefore not possible.

3) If the only fault occurs in measuring p_5.

- In the circuit of measuring p_5, only faults after CX in the middle can lead to non-trivial 2-qubit errors Z_4Z_5 and Y_4Z_5. These errors both commute with $p_3 = XZIIZ$ and $p_4 = ZXZII$, i.e., both M_3 and M_4 are fixed to be +1. The syndromes in Eq. (S7) and Eq. (S8) are thus not possible.

- If the only fault occurs on an idling location of the data qubits C1 or C2, the measurement of p_5 is then correct. Because the induced single-qubit errors commute with $p_5 = IIZXZ$, which means the Pauli correction fixes M_5 to be +1. The syndrome in Eq. (S7) are therefore not possible. In addition, any single-qubit error on C1 or C2 cannot anti-commute with $p_1 = IZXZI$, $p_2 = ZIIZX$, $p_3 = XZIIZ$ and $p_4 = ZXZII$ at the same time, the syndrome in Eq. (S8) is also excluded.

- Up to a stabilizer or a logical X operator, other single faults can only induce a single-qubit error on the data qubits C3, C4 or C5. Such single-qubit errors cannot anti-commute with $p_3 = XZIIZ$ and $p_4 = ZXZII$ at the same time, the syndromes in Eq. (S7) and Eq. (S8) are therefore not possible.

VII. ERROR DISTRIBUTION IN THE PREPARED STATE

The overlaps between the prepared state ρ and the states $E\left|-\right\rangle_L$, $E\left|+\right\rangle_L$, with E identity or a single-qubit error ($P_{0,-}$, $P_{1,-}$, $P_{0,+}$, $P_{1,+}$) can be expressed in terms of the measured 31 expectation values as

$$\begin{aligned}P_{0,-} =& \mathrm{Tr}(\left|-\right\rangle_L \left\langle-\right|_L \rho)\\ =& \frac{1}{32}(1 + \langle IZXZI\rangle + \langle ZIIZX\rangle + \langle XZIIZ\rangle + \langle ZXZII\rangle + \langle IIZXZ\rangle + \langle YIXIY\rangle\\ &+ \langle IYYIX\rangle + \langle XIYYI\rangle + \langle IXIYY\rangle + \langle YYIXI\rangle - \langle ZZYXY\rangle - \langle YXYZZ\rangle\\ &- \langle ZYXYZ\rangle - \langle XYZZY\rangle - \langle YZZYX\rangle - \langle XXXXX\rangle + \langle IXZZX\rangle + \langle IYXXY\rangle\\ &+ \langle IZYYZ\rangle + \langle XIXZZ\rangle + \langle XXYIY\rangle + \langle XYIYX\rangle + \langle XZZXI\rangle + \langle YIYXX\rangle\\ &+ \langle YXXYI\rangle + \langle YYZIZ\rangle + \langle YZIZY\rangle + \langle ZIZYY\rangle + \langle ZXIXZ\rangle + \langle ZYYZI\rangle\\ &+ \langle ZZXIX\rangle),\end{aligned} \tag{S9}$$

$$\begin{aligned}P_{1,-} =& \sum_{E\in\mathcal{E}} \mathrm{Tr}(E\left|-\right\rangle_L \left\langle-\right|_L E\,\rho)\\ =& \frac{1}{32}(15 + 3\langle IZXZI\rangle + 3\langle ZIIZX\rangle + 3\langle XZIIZ\rangle + 3\langle ZXZII\rangle + 3\langle IIZXZ\rangle + 3\langle YIXIY\rangle\\ &+ 3\langle IYYIX\rangle + 3\langle XIYYI\rangle + 3\langle IXIYY\rangle + 3\langle YYIXI\rangle + 5\langle ZZYXY\rangle + 5\langle YXYZZ\rangle\\ &+ 5\langle ZYXYZ\rangle + 5\langle XYZZY\rangle + 5\langle YZZYX\rangle + 5\langle XXXXX\rangle - \langle IXZZX\rangle - \langle IYXXY\rangle\\ &- \langle IZYYZ\rangle - \langle XIXZZ\rangle - \langle XXYIY\rangle - \langle XYIYX\rangle - \langle XZZXI\rangle - \langle YIYXX\rangle\\ &- \langle YXXYI\rangle - \langle YYZIZ\rangle - \langle YZIZY\rangle - \langle ZIZYY\rangle - \langle ZXIXZ\rangle - \langle ZYYZI\rangle\\ &- \langle ZZXIX\rangle),\end{aligned} \tag{S10}$$

$$\begin{aligned}P_{0,+} =& \mathrm{Tr}(\left|+\right\rangle_L \left\langle+\right|_L \rho)\\ =& \frac{1}{32}(1 - \langle IZXZI\rangle - \langle ZIIZX\rangle - \langle XZIIZ\rangle - \langle ZXZII\rangle - \langle IIZXZ\rangle - \langle YIXIY\rangle\\ &- \langle IYYIX\rangle - \langle XIYYI\rangle - \langle IXIYY\rangle - \langle YYIXI\rangle + \langle ZZYXY\rangle + \langle YXYZZ\rangle\\ &+ \langle ZYXYZ\rangle + \langle XYZZY\rangle + \langle YZZYX\rangle + \langle XXXXX\rangle + \langle IXZZX\rangle + \langle IYXXY\rangle\\ &+ \langle IZYYZ\rangle + \langle XIXZZ\rangle + \langle XXYIY\rangle + \langle XYIYX\rangle + \langle XZZXI\rangle + \langle YIYXX\rangle\\ &+ \langle YXXYI\rangle + \langle YYZIZ\rangle + \langle YZIZY\rangle + \langle ZIZYY\rangle + \langle ZXIXZ\rangle + \langle ZYYZI\rangle\\ &+ \langle ZZXIX\rangle),\end{aligned} \tag{S11}$$

$$
\begin{aligned}
P_{1,+} &= \sum_{E\in\mathcal{E}} \mathrm{Tr}(E\,|+\rangle_L\,\langle+|_L\,E\,\rho) \\
&= \frac{1}{32}(15 - 3\langle IZXZI\rangle - 3\langle ZIIZX\rangle - 3\langle XZIIZ\rangle - 3\langle ZXZII\rangle - 3\langle IIZXZ\rangle - 3\langle YIXIY\rangle \\
&\quad - 3\langle IYYIX\rangle - 3\langle XIYYI\rangle - 3\langle IXIYY\rangle - 3\langle YYIXI\rangle - 5\langle ZZYXY\rangle - 5\langle YXYZZ\rangle \\
&\quad - 5\langle ZYXYZ\rangle - 5\langle XYZZY\rangle - 5\langle YZZYX\rangle - 5\langle XXXXX\rangle - \langle IXZZX\rangle - \langle IYXXY\rangle \\
&\quad - \langle IZYYZ\rangle - \langle XIXZZ\rangle - \langle XXYIY\rangle - \langle XYIYX\rangle - \langle XZZXI\rangle - \langle YIYXX\rangle \\
&\quad - \langle YXXYI\rangle - \langle YYZIZ\rangle - \langle YZIZY\rangle - \langle ZIZYY\rangle - \langle ZXIXZ\rangle - \langle ZYYZI\rangle \\
&\quad - \langle ZZXIX\rangle).
\end{aligned} \tag{S12}
$$

VIII. PSEUDOCODE

In this section we provide pseudocodes (Python based) for compiling quantum circuits (in our native gate scheme) into the underlying pulse sequences.

Pseudocode 1. A dictionary containing the relevant parameters for all qubits.

```
gate_params={
# Electron spin qubit (auxiliary qubit)
'ms-1_frq': 1.746666e9, # Frequency of the ms = 0 to ms = -1 transition (the electron spin qubit frequency)
'ms+1_frq': 4.008650e9, # Frequency of the ms = 0 to ms = +1 transition
'Hermite_pi_length':  168e-9, # Hermite t_pulse  parameter in seconds for the microwave pi pulse (see Methods for definition)
'Hermite_pi_amp': 0.733, # Microwave amplitude used for electron pi pulses
'Hermite_pi2_length': 100e-9, # Hermite t_pulse  parameter in seconds for the microwave pi/2 pulse (see Methods for definition)
'Hermite_pi2_amp': 0.478,

# Qubit #1 (C13 #1)
'q1_freq_0': 431956, # The precession frequency for the ms = 0 electron spin projection (Hz)
'q1_freq_m1': 469020, # The precession frequency for the ms = -1 electron spin projection (Hz)

# Two-qubit gate parameters
'q1_tau': [7.218e-6], # 2tau is the interpulse delay in the dynamical decoupling gate sequence
'q1_N': [44], #Total number of pulses for the gate
'q1_extra_phase_correction': np.array([]), # A vector of the phase correction list for the other qubits when applying a DD (or DDRF) gate on C1 (calibration routine described in Supplementary Fig. 7)
'q1_DDRF': False, # whether the dynamical decoupling sequence is interleaved with RF pulses (True) or not (False)
'q1_RF_freq': 469043.152, # The RF frequency used for the RF pulse or DDRF gate if used (Hz)

# Single qubit gate parameters
'q1_pi_length_RF': 1.2e-3, #Length of the RF pi pulse on the nuclear spin (seconds)
'q1_pi2_length_RF': 0.6e-3, #Length of the RF pi/2 pulse on the nuclear spin (seconds)
'q1_RF_amp': 2.04,  # The RF amplitude used for RF pulses (single qubit pulses)
'q1_extra_phase_correction_list_RF' : np.array([]) #A vector of the phase correction list for the other qubits when applying an RF pi pulse on qubit 1

# Qubit #2 (C13 #2)
...
# Qubit #3 (C13 #3)
...
# Qubit #4 (C13 #4)
...
# Qubit #5 (C13 #5)
...
# Qubit #6 (N14, m_I = {0,+1} basis, flag qubit, treated analogously to C13 qubits)
...
}
```

Pseudocode 2. Tracking qubits phases.

```

def calculate_c_phases(phases_before, gate_length, el_state = '-1', gate_type = None):
'''
Pseudocode for calculating and tracking the phases of the nuclear-spin qubits (C13 and N14 nuclear spin qubits).

Args:
phases_before: a vector of the phases of the nuclear-spin qubits before applying the gate (over which the phases need to be tracked)
gate_length: the evolution time of the gate over which the phases need to be tracked
el_state: is the electron spin state during the evolution, from {-1, 0, 'sup'}, where 'sup' is any superposition state
gate_type : default is None. In the case of 'DD_gate' or 'DDRF_gate', the two-qubit gate crossphase is accounted for

Returns:
phases_after: a vector of the phases of the nuclear-spin qubits after applying the gate

'''

    phases_after = np.zeros(len(phases_before))

    # Calculating the qubit phases depending on the electron spin state
    for j in range(len(phases_before)):
        if el_state == '-1':
                phases_after[j] = phases_before[j] + gate_params['q'+str(j) + '_freq_m1']*gate_length*360
                # If the electron spin is in a superposition state, it is automatically dynamically decoupled, and so the evolution frequency of C13 nuclear spins is  the average of freq-0 (their evolution frequency for ms = 0) and freq-m1 (their evolution frequency for ms = -1)
        elif el_state == 'sup':
                phases_after[j] = phases_before[j] + ((gate_params['q'+str(j)+'_freq_m1'] + gate_params['q'+str(j) + '_freq_0'])/2.) * gate_length*360
        else:
                phases_after[j] = phases_before[j] + gate_params['q'+str(j)+'_freq_0']*gate_length*360

        # Adding the extra phase corrections due to the application of the DD or DDRF gate (see Supplementary Fig. 7 for explanation)
        if gate_type == 'DD_gate' or 'DDRF_gate':
                phases_after += gate_params['q'+str(j)+'_extra_phase_correction']

    return phases_after%360
```

Pseudocode 3. Phase synchronization and control.

```

def insert_phase_shift_gate(seq, qubit_nr, phases_before, target_phase = 0, el_state = '-1'):
'''
This pseudocode calculates the required phase-shift gate parameters to account for phase acquired during other operations and apply any desired single-qubit Z rotations (see Supplementary Fig. 5).
Args:
seq: the main gate sequence (for which a phase-shift gate might be added)
qubit_nr: the index of the nuclear-spin qubit targeted in the next operation, for which a phase-shift gate might be needed
target_phase: the required C13 phase for which a phase-shift gate might be needed
phases_before: a vector of the phases of the C13 spins involved in the experiment before applying the phase gate
el_state: the electron spin state during the evolution
Returns:
c_phase_after: a vector of the phases of the C13 spins involved in the experiment after applying the phase-shift gate
seq
'''
    global seq
    # Calculating the difference between the target phase and the actual phase before the phase-shift gate
    if target_phase > phases_before[qubit_nr]%360:
        extra_phase = (target_phase - phases_before[qubit_nr])%360
    else:
        extra_phase = (target_phase + 360 - phases_before[qubit_nr])%360

    # Setting the phase-shift gate parameters to be used depending on the electron spin state '-1','0', or 'sup' (superposition)
    #If the electron spin is in an eigenstate ('0' or '-1') the phase-shift gate is done by wait times (as the electron T1 is > 1hour)
    if el_state == '-1':
        phase_gate_length = (extra_phase)*(pi/180) / (2*pi*gate_params['q'+str(qubit_nr) + '_freq_m1'])
        seq = wait(seq,phase_gate_length)
    elif el_state == '0':
        phase_gate_length = (extra_phase)*(pi/180) / (2*pi*gate_params['q'+str(qubit_nr) + '_freq_0'])
        seq = wait(seq, phase_gate_length)

    #If the electron spin is in a superposition state, the phase-shift gate is done by applying a four pulse dynamical decoupling sequence (XY4) to mitigate the electron spin decoherence
    elif el_state == 'sup':
        sup_freq = (2*pi*gate_params['q'+str(qubit_nr) + '_freq_m1'] + 2*pi*gate_params['q'+str(qubit_nr) + '_freq_0'])/2. # The frequency is the average of ms = 0 and ms = -1 frequencies
        min_decoupling_duration = 8*gate_params[    Hermite_pi_length    ] # minimum decoupling duration is set by the pi pulse duration and number of pulses (2*N*tau_min), tau_min is the length of the electron pi pulse.

        phase_gate_length = (extra_phase)*(pi/180) / sup_freq

       # Adding a 2*pi phase until the phase-shift gate length is more than the minimum decoupling length
        while phase_gate_length <= min_decoupling_duration:
           extra_phase + = 2*pi
           phase_gate_length = (extra_phase)*(pi/180) / sup_freq

        dec_tau = phase_gate_length/8. # Calculating tau for the decoupling sequence (length = 2*N*tau, N = 4)

        # Applying the four pulse decoupling sequence - note that the pi pulse duration is accounted for when generating the AWG pulse sequence as shown in Supplementary Fig. 5
        Apply_dd_sequence (N=4, tau= dec_tau)

    phases_after = calculate_c_phases(phases_before, phase_gate_length, el_state = el_state)%360

    return phases_after
```

Pseudocode 4. Applying two-qubit gates.

```
def apply_two_qubit_gate(seq, qubit_nr, phases_before, gate_phase):
'''
This function is used to apply the dynamical decoupling two-qubit gate on a target
  nuclear-spin qubit
Args:
seq: the main sequence (to which the two-qubit gate will be added)
qubit_nr: the index of the nuclear-spin qubit being targeted by the two-qubit gate
phases_before: a vector of the phases of the C13 spins involved in the experiment
  before applying the DD gate
gate_phase: the required C13 phase before applying the dynamical decoupling
  sequence which sets the target rotation axis of the gate as explained in
  Supplementary Figs. 4, 5.
Returns:
phases_updated: a vector of the phases of the C13 spins involved in the experiment
  after applying the two-qubit gate
'''
    global seq

    # Deciding the type of two-qubit gate (DDRF or DD) depending on the targeted
      C13 nuclear spin
    DDRF = gate_params['q'+str(qubit_nr) + '_DDRF']
    # Getting the relevant gate parameters depending on the gate type (DDRF or DD):
    # N (number of pulses)
    # tau (half of interpulse delay)
    # RF_frequency(if DDRF gate)
    # RF_amp (if DDRF gate, the RF amplitude used)
    N = gate_params['q'+str(qubit_nr) + '_N'][0]
    tau = gate_params['q'+str(qubit_nr) + '_tau'][0]

    if DDRF:
        gate_type = 'DDRF_gate'
        RF_freq = gate_params['q'+str(qubit_nr) +  'RF_freq']
        RF_amp = gate_params['q'+str(qubit_nr) +  'RF_amp']
    else:
        gate_type = 'DD_gate'

    # Insert a phase-shift gate to adjust the target C13 phase
    If gate_phase != phases_before[qubit_nr]:
        phases_updated = insert_phase_shift_gate(seq, qubit_nr, phases_before,
          gate_phase, el_state = 'sup')

    if DDRF:    # Adding a DDRF two-qubit gate
        apply_nuclear_RF_gate(N, tau, RF_freq, RF_amplitude)
    else:       # Adding a DD two-qubit gate
        apply_nuclear_gate(N,tau)

    # Updating the C13 phases to account for the applied DD or DDRF gate sequence
    gate_length = 2*N*tau  # gate length to be used in phase tracking
    phases_updated = calculate_c_phases(phases_before, gate_length, el_state =
      'sup', gate_type = gate_type)%360

    return phases_updated
```

Pseudocode 5. Data qubit initialization.

```
def initialize_qubits(seq,qubit_nr_list,phases_before):
'''
This pseudocode is used to generate the gate sequence for initializing the C13 nuclear spins (as illustrated in Supplementary Fig. 6)
Args:
seq: the main gate sequence
qubit_nr_list: the index of the C13 qubits to be initialized
phases_before: a vector of the phases of the nuclear-spin qubits involved in the experiment before applying the initialization sequence

Returns:
phases_updated: a vector of the phases of the nuclear-spin qubits involved in the experiment after applying the initialization sequence
'''
    global seq
    for j, qubit_nr in enumerate(qubit_nr_list):

        electron_pi2_pulse(seq, phase = 90) # applying a pi/2 pulse on the NV electron spin (along Y axis)

        phases_before[qubit_nr] = 0. #setting the initial phase to zero (the qubit phase at this point doesn't matter)

        # Applying a two-qubit gate (along x axis) on the target C13 nuclear spin
        phases_updated = apply_two_qubit_gate(seq, qubit_nr, phases_before, gate_phase=0)
        electron_pi2_pulse(seq,phase = 0) # applying a pi/2 pulse on the NV electron spin (along x axis)

        # Applying a two-qubit gate (along -y axis) on the target C13 nuclear spin
        phases_updated = apply_two_qubit_gate(seq, qubit_nr, phases_updated, gate_phase=90)

        phases_updated = electron_reset(seq) # Applying an electron reset element (optical repumping)

        return phases_updated%360
```

Pseudocode 6. Performing a stabilizer measurement.

```python

def stabilizer_msmt_sequence(seq, qubit_nr_list, phases_before, stabilizer_basis = ['X', 'X'], el_state_in = '0', final_pi2_phase = 0):

'''
This pseudocode is used to generate the gate (pulse) sequence used to perform a stabilizer measurement (defined in our native gate scheme - e.g Supplementary Fig. 4, 6). See Ref. 1 for more details on performing stabilizer measurements using our native gates and how to account for the extra basis rotations.

Args:
seq: the main gate sequence (to which the stabilizer measurement sequence will be added)
qubit_nr_list: the index of the nuclear-spin qubits involved in the experiment
phases_before: a vector of the phases of the nuclear-spin qubits involved in the experiment before applying the stabilizer measurement sequence
stabilizer_basis: the Pauli operations of the stabilizer measurement
el_state_in: the electron spin state before the stabilizer measurement (this matters for the phase of the initial pi/2 pulse)
final_pi2_phase: the phase of the final pi/2 pulse on the electron

Returns:
phases_updated: a vector of the phases of the nuclear-spin qubits involved in the experiment after applying the stabilizer sequence
'''

    global seq

    # Adding the initial pi/2 pulse - the phase of the pulse depends on the initial electron spin state ('0' or '-1')
    if el_state_in == '0':
      electron_pi2_pulse (seq, phase = 90)
    else:
      electron_pi2_pulse (seq, phase = 270)

    # Setting the gate_phase according to the targeted Pauli operation (as explained in Pseudocode 3). Note that stabilizer_basis along Z are normally compiled to Y basis in our native gate scheme as shown in Supplementary Fig. 4.
    for j, qubit_nr in enumerate(qubit_nr_list):
        if stabilizer_basis[j] != 'I':
            if stabilizer_basis[j] == 'X':
                gate_phase = 0
            elif stabilizer_basis[j] == 'Y':
                gate_phase = 270
            if stabilizer_basis[j] == '-X':
                gate_phase = 180
            elif stabilizer_basis[j] == '-Y':
                gate_phase = 90

    # Applying a DD two-qubit gate on the target C13 nuclear spin with the phase defined by the parity axis as above
    phases_updated = apply_two_qubit_gate(seq, qubit_nr, phases_before, gate_phase)

    # Adding the final electron pi/2 pulse. The phase depends on the specific circuit compilation (e.g. Supplementary Fig. 8).
    electron_pi2_pulse (seq, pi2_phase) # Applying the final electron pi/2 pulse

    phases_updated = Readout_parity_element(seq, phases_updated) # Adding the electron readout element for the parity measurement

  return phases_updated%360
```

Pseudocode 7. Measuring multi-qubit operators of the data qubits (data qubits readout).

```

def readout_qubits(seq,qubit_nr_list,phases_before,RO_basis = ['X'],el_state_in = '-1'):
'''
This code is used to generate the gate sequence required for measuring a multi-qubit operator of the data qubits (as illustrated in Supplementary Fig. 6)

Args:
seq: the main gate sequence (to which the readout sequence will be added)
qubit_nr_list: the index of the nuclear-spin qubits involved in the experiment
phases_before: a vector of the phases of the nuclear-spin qubits involved in the experiment before applying the readout sequence
RO_basis: the basis of the readout measurement (e.g. RO_basis for measuring the operator XXIXI is ['X','X','I','X','I'])
el_state_in: the electron spin state before the readout sequence (this matters for the phase of the initial pi/2 pulse)

Returns:
c_phases_: a vector of the phases of the nuclear-spin qubits involved in the experiment after applying the stabilizer sequence
'''

    global seq

    if el_state_in == '0': # Invert the electron spin state to -1 before readout (required for selective RF pulses )
        electron_pi_pulse (seq, phase = 0)

    # If readout in the Z-basis, we apply an RF pulse along -x (as explained in Supp Fig. 6) to map Z-basis to Y-basis
    for j, qubit_nr in enumerate(qubit_nr_list):
        if RO_basis[j] == 'Z':
                phases_updated = apply_RF_pi2_pulse(seq, qubit_nr, phases_before, phase = 180 )

    # Adding the initial pi/2 pulse - the phase of the pulse depends on the initial electron spin state ('0' or '-1')
    electron_pi_pulse (seq, phase = 270)

    nonidentities = 0
    for j, qubit_nr in enumerate(qubit_nr_list):
        if stabilizer_basis[j] != 'I':
            nonidentities += 1
            if RO_basis[j] == 'X':
                gate_phase = 0
            elif RO_basis[j] == 'Y':
                gate_phase = 270
            elif RO_basis[j] == 'Z':
                gate_phase = 90 # Z-basis is done by mapping it to the Y-basis (Supplementary Fig. 6)
            else:
                gate_phase = RO_basis[j] # the phase should be directly passed if not one of the options above

    # Applying a DD two-qubit gate on the target C13 nuclear spin with the phase defined by the RO basis as above
    phases_updated = apply_two_qubit_gate(seq, qubit_nr, phases_before, gate_phase)

    # The phase of the final pi/2 pulse depends on the number of two-qubit gates (non-identities) involved in the readout as explained in Supplementary Fig. 6
    pi2_phase = (((nonidentities%4)-1)*90.)%360.

    electron_pi2_pulse (seq, pi2_phase) #Applying the final electron pi/2 pulse
    Readout_electron(seq, phases_updated) # Adding the final NV electron readout
    return
```

Pseudocode 8. Example code for generating a 4-qubit GHZ state

```
# Example code for preparing and characterizing a 4-qubit GHZ state using a conditional stabilizer measurement

qubit_list = [1,2,3,4] # defining which qubits to be used
RO_basis_list = [] # The 15 operators (that define the prepared 4-qubit GHZ state) to be measured

For i in range (RO_basis_list):
    seq = []
    initialize_NV (seq) # Preparing the NV in the negative charge, bringing it in resonance with the lasers and then initializing it in the ms = 0 state (as shown in Supplementary Fig. 3)

    initialize_Nitrogen(seq) #Initializing the N14 nuclear-spin into mI = 0 state as shown in Supplementary Fig. 6

    phases_before = np.array([0 for j in range(28)]) #This normally includes all 27 spins qubits of the processor (in addition to the N14 spin - q0 here)

    phases_updated = init_qubits((seq,qubit_list, phases_before) #Initializing the data qubits (here C13 nuclear spin qubits 1,2,3,4)

    phases_updated = stabilizer_msmt_sequence(seq, qubit_list, phases_updated, stabilizer_basis = ['X', 'X', 'X', 'X'], el_state_in = '0', final_pi2_phase = '-Y') #Applying the XXXX stabilizer measurement. (Note that in this example the stabilizer measurement is done in a conditional way (we only continue upon measuring the electron state to be in the ms = 0 state). However, this part is Handled by the micro-controller (real-time control) and not included in this pseudocode which is mainly for the AWG pulse sequences.)

    readout_qubits(seq, qubit_list, phases_updated, RO_basis_list[i], el_state_in = '0') #Reading out the data qubits

    Generate_AWG_seq(seq) #This translates the pulse sequence into the instructions for the AWG. This step is strongly hardware dependent.
```


[1] Abobeih, M. *From atomic-scale imaging to quantum fault-tolerance with spins in diamond.* Ph.D. thesis, Delft University of Technology (2021).

[2] Doherty, M. *et al.* Theory of the ground-state spin of the NV-center in diamond. *Phys. Rev. B* **85**, 205203 (2012).

[3] Abobeih, M. H. *et al.* Atomic-scale imaging of a 27-nuclear-spin cluster using a quantum sensor. *Nature* **576**, 411–415 (2019).

[4] Abobeih, M. H. *et al.* One-second coherence for a single electron spin coupled to a multi-qubit nuclear-spin environment. *Nat. Commun.* **9**, 2552 (2018).

[5] Robledo, L. *et al.* High-fidelity projective read-out of a solid-state spin quantum register. *Nature* **477**, 574–578 (2011).

[6] Taminiau, T. H., Cramer, J., Sar, T. v. d., Dobrovitski, V. V. & Hanson, R. Universal control and error correction in multi-qubit spin registers in diamond. *Nat. Nanotech.* **9**, 171–176 (2014).

[7] Bradley, C. E. *et al.* A ten-qubit solid-state spin register with quantum memory up to one minute. *Phys. Rev. X* **9**, 031045 (2019).

[8] Warren, W. S. Effects of arbitrary laser or NMR pulse shapes on population inversion and coherence. *J. Chem. Phys* **81**, 5437–5448 (1984).

[9] Johansson, J. R., Nation, P. D. & Nori, F. Qutip: An open-source python framework for the dynamics of open quantum systems. *Computer Physics Communications* **183**, 1760–1772 (2012).

[10] Chao, R. & Reichardt, B. W. Quantum error correction with only two extra qubits. *Phys. Rev. Lett.* **121**, 050502 (2018).

[11] Chamberland, C. & Beverland, M. E. Flag fault-tolerant error correction with arbitrary distance codes. *Quantum* **2**, 53 (2018).

[12] Aliferis, P., Gottesman, D. & Preskill, J. Quantum accuracy threshold for concatenated distance-3 code. *Quantum Inf. Comput.* **6**, 97–165 (2006).

[13] Chao, R. & Reichardt, B. W. Flag fault-tolerant error correction for any stabilizer code. *PRX Quantum* **1**, 010302 (2020).
